# How did COVID-19 case distribution associate with the urban built environment? A community-level exploration in Shanghai focusing on non-linear relationship

**DOI:** 10.1371/journal.pone.0309019

**Published:** 2024-10-16

**Authors:** Jingyi Gao, Yifu Ge, Osamu Murao, Yitong Dong, Guofang Zhai

**Affiliations:** 1 Department of Architecture and Building Science, Graduate School of Engineering, Tohoku University, Sendai, Japan; 2 School of Architecture and Urban Planning, Nanjing University, Nanjing, China; 3 International Research Institute of Disaster Science, Tohoku University, Sendai, Japan; 4 Shanghai Urban Planning and Design Co., Ltd. of Shanghai Planning Institute, Shanghai, China; Universiti Kebangsaan Malaysia, MALAYSIA

## Abstract

Several associations between the built environment and COVID-19 case distribution have been identified in previous studies. However, few studies have explored the non-linear associations between the built environment and COVID-19 at the community level. This study employed the March 2022 Shanghai COVID-19 pandemic as a case study to examine the association between built-environment characteristics and the incidence of COVID-19. A non-linear modeling approach, namely the boosted regression tree model, was used to investigate this relationship. A multi-scale study was conducted at the community level based on buffers of 5-minute, 10-minute, and 15-minute walking distances. The main findings are as follows: (1) Relationships between built environment variables and COVID-19 case distribution vary across scales of analysis at the neighborhood level. (2) Significant non-linear associations exist between built-environment characteristics and COVID-19 case distribution at different scales. Population, housing price, normalized difference vegetation index, Shannon’s diversity index, number of bus stops, floor–area ratio, and distance from the city center played important roles at different scales. These non-linear results provide a more refined reference for pandemic responses at different scales from an urban planning perspective and offer useful recommendations for a sustainable COVID-19 post-pandemic response.

## 1. Introduction

On May 5, 2023, the World Health Organization (WHO) declared that COVID-19 was no longer a public health emergency of international concern [1]. This indicates that the global community has entered the post-pandemic era. According to the WHO’s updated Strategic Preparedness and Response Plan for 2023–2025, national responses to COVID-19 should shift from emergency measures to long-term management, making the urban environment more resilient, equal, diverse, and sustainable [2]. The associations between COVID-19 case distribution and the urban environment have become a subject of interest for urban planners.

The neighborhood built environment is recognized as a key environmental factor affecting the public health [3, 4]. It is the major environmental context influencing the COVID-19 case distribution. Researchers often delineate buffer zones at specific scales and use them as spatial representations of residents’ activity areas. Previously, buffer zones with radii ranging from 50 m to 5 km have been used in different studies [5, 6]. This variability can result in the strength and direction of correlations between variables changing depending on the spatial scale of analysis. This issue is known as the modifiable areal unit problem (MAUP) [7]. According to Openshaw (1996) [8], the solution to the MAUP is to identify appropriate geospatial scales and geographic unit divisions for specific studies. Additionally, there may be scaling effects between COVID-19 case distribution and the neighborhood built environment that have not been identified. Therefore, an analysis based on different spatial scales is necessary.

Many studies of the built environment and COVID-19 spread tend to predefine the relationship as linear, which potentially misestimating or oversimplifying complex associations [9]. Most studies have used linear models, such as multivariable linear regression [10], generalized linear models [11, 12], weighted least squares (LS) [13], hierarchical multiple regression [14], and ordinary least squares (OLS) [12, 15]. These models can only produce linear results, which has limitations. Previous linear models have indicated a notable correlation between the built environment and the spread of COVID-19. However, Luo et al. identified a significant nonlinear relationship between the built environment and the spread of COVID-19 in the United States. This relationship exhibited a significant threshold effect, and the linear model proved inadequate for accurately estimating the spatial variability of this nonlinear relationship [16]. Similarly, Ma et al.’s investigation into the relationship between the built environment and the spread of COVID-19 in Chinese townships found that the density of gyms and sports centers had the greatest impact at 2/km², with the effect remaining constant with further increases in density [17]. In this context, identifying non-linear results might provide more specific urban planning guidance compared to linear results. Prior studies of the built environment and COVID-19 are further discussed in Section 2.

Considering the lack of research on non-linear relationships at different scales, this study focused on the community level and explored the following two research questions.

1. Do relationships between built environment variables and COVID-19 case distribution vary across scales of analysis?
2. What are the non-linear relationships between built-environment characteristics and COVID-19 case distribution at different scales?

## 2. Previous studies on the COVID-19 case distribution associated with the built environment

Factors influencing COVID-19 case distribution in urban areas have been explored in previous studies. For example, population density has been incorporated as an important indicator in research related to COVID-19 spread in various countries [12, 14, 15, 18]. High population density [13] and increased air pollution caused by excessive population [9] are often correlated with the rapid spread of the pandemic [19]. Additionally, researchers often associate socio-economic factors with spatial location. Differences in economic development levels have been regarded as exacerbating the spread risk of COVID-19 [20]. For instance, researchers identified the key role of economic activities in COVID-19 transmission in Italy. More productive areas with intensive economic activities (e.g., a higher percentage of employment in manufacturing) have higher possibilities of triggering COVID-19 spread [21]. A study that considered variables including the number of visitors, tourists staying overnight, and the generated income from their activities found a significant correlation between the number of cases and these economic factors [22]. Several scholars believe that COVID-19 spread is positively correlated with gross domestic product (GDP) [10].

Urban-planning attributes, including architecture, urban density, land use, and landscape, have also been found to correlate with COVID-19 case distribution [23–25]. The significance of greenspace is frequently discussed, though its impact varies in different studies. For example, Kwok et al. found no significant relationship between greenspace and COVID-19 cases in Hong Kong [26]. Conversely, greenspace was associated with a reduced risk of COVID-19 mortality in the United States [27]. Although scholars hold different views on the relationship between COVID-19 cases and urban-density variables, the density indicators selected by researchers have common features, such as road density [28]; floor–area ratio (FAR), which characterizes building density [29]; and the number of points of interest (POIs), representing the facility density [30]. Regarding land use, the Shannon diversity index (SHDI) is commonly used to characterize land-use mixtures. A study conducted in Melbourne, Australia, analyzed the geographic distribution of COVID-19 cases and its correlation with built-environment variables. This study validated and highlighted the importance of considering the diversity of the built environment and mixed-use development when investigating the geographical distribution of COVID-19 cases within a city [31].

The aforementioned research provides a theoretical basis for selecting variables in this study and inspires the methodological approach. In previous studies, conventional spatial analysis models were predominantly used. Many studies first employed spatial autocorrelation analysis to obtain global spatial autocorrelation (Moran’s I) and local indicators of spatial association to measure the spatial distribution characteristics of cases [10, 13, 31–33]. Subsequently, statistical methods such as Spearman or Pearson’s were used to test the relationship between the selected indicators and the dependent variable [15, 18, 19, 22]. Finally, a spatial regression model was constructed [34]. Compared to conventional regression models, some scholars have used use machine learning methods to obtain more accurate results in studying COVID-19. This includes using the elastic net machine-learning algorithm to optimize conventional linear models for predicting and verifying the significance of variables or using the multilayer-perceptron model to model and predict the number of cases [35]. However, the objective non-linear relationship between variables and dependent variables has not been well identified, which may lead to the ignoration of spatial attribute thresholds. Ignoring the spatial thresholds reflected by non-linear associations may further cause biased perceptions and the formulation of incorrect policies [36, 37].

Considering the community transmission characteristics of COVID-19, communities must be prepared for COVID-19 challenges [38]. In 2020, experts urged countries worldwide to strengthen monitoring of community COVID-19 infection and changing trends [39]. Compared to the early days of the COVID-19 pandemic, controlling community transmission to reduce the risk of infection was the top priority. In recent years, as the COVID-19 pandemic has subsided, the focus of community preparation in the post-pandemic era has shifted to making communities more resilient and sustainable [40]. Many studies have examined COVID-19 case distribution at the community level [41–46]. Although these studies provide a rationale for community-based COVID-19 research, they are mostly large-scale, involving nations, regions, cities, and districts/counties. Small-scale studies focusing on communities, scale differences and the non-linear relationship between the built environment and COVID-19 case distribution are still insufficient.

## 3. Methods

The COVID-19 pandemic outbreak in Shanghai in March 2022 was selected as a case study, and officially announced COVID-19 addresses were collected. Built-environment variables associated with COVID-19 spread were selected based on a literature review. We used multi-source data to characterize the built environment and constructed a database combining the collected COVID-19 addresses at the community level. BRT models were developed to explore the non-linear associations between built-environment characteristics and COVID-19 case distribution. In this section, we will sequentially outline the variable selection, the collection and processing of COVID-19 and community data, and the steps involved in BRT model construction.

### 3.1 Data collection and processing

#### 3.1.1 Data collection and variable selection

Shanghai, a mega-city in China, was selected as the site for this case study. It has a high population and population density, which potentially increase human contact and viral transmission. A large-scale COVID-19 outbreak occurred in Shanghai in March 2022. Initially, Shanghai did not implement strict measures such as lockdowns to control the spread of the pandemic. The period selected for this study was from May 5 to May 31, a time when official data on case reports were available, and strict control measures had not yet been implemented.

Data reflecting the COVID-19 case distribution in Shanghai were collected from the daily pandemic information released by the Shanghai Municipal Health Commission (https://wsjkw.sh.gov.cn/yqtb/), which included the number of infected individuals and their residential addresses. Community data, including names and addresses for buffer zone construction in the main urban area were retrieved from Shanghai Bendibao (http://sh.bendibao.com/), a platform that publishes local information.

Based on review of previous studies in built environments [47, 48] and COVID-19-related research [25, 49], a total of four categories covering 18 variables were determined for this study. The first category focused on facilities closely related to residents’ lives, including various POIs and bus stops [50, 51]. The second category addressed density factors such as road density population density, and bus line density [50, 52–55]. The third category encompassed land use characteristics, including land-use mixture (represented by Shannon’s Diversity Index), Normalized Difference Vegetation Index (NDVI), Floor–Area Ratio (FAR), and distance from the city center [56–59]. The fourth category included socio-economic factors, specifically housing prices [50]. Data necessary for calculating these variables were collected. Detailed descriptions of each variable and their original data sources are provided in Table 1. Given the correlation of these variables with COVID-19 case distribution, we hypothesized differences in their contributions to the number of COVID-19 addresses across different scales of analysis. Non-linear findings can elucidate their associations with COVID-19 case distribution at various scales.

**Table 1:** Variables and data sources of the built environment.

Category	Variable	Description	Data Source
Distribution of facilities	(X1) POI of dining & gourmet	Number of dining & gourmet facilities in the buffer	WeChat official account: Dixuedashuju (In Chinese) https://mp.weixin.qq.com/s/zYwF3a8RI-APVT7sMXp5uQ
	(X2) POI of shopping	Number of shopping facilities in the buffer	
	(X3) POI of financial institutions	Number of financial institutions in the buffer	
	(X4) POI of science and education facilities	Number of science and education facilities in the buffer	
	(X5) POI of tourist attractions	Number of tourist attraction facilities in the buffer	
	(X6) POI of domestic services	Number of domestic service facilities in the buffer	
	(X7) POI of leisure & entertainment	Number of leisure & entertainment facilities in the buffer	
	(X8) POI of medical & healthcare	Number of medical & healthcare facilities in the buffer	
	(X9) POI of sports & fitness	Number of sports & fitness facilities in the buffer	
	(X10) Number of bus stops	Number of bus stops in the buffer	WeChat official account: Lifangshujuxueshe (In Chinese) https://mp.weixin.qq.com/s/hafU51Zqj5ig_sjgY78jRg The original data was claimed from an open-source bus information query website.
Density	(X11) Road density	The density of roads in the buffer (km/km^2^)	Open Street Map (OSM) The density was calculated by the authors of this study.
	(X12) Population density	The density of the population in the buffer (/km^2^)	WorldPop The population of the grid Population counts/constrained individual countries 2020 UN adjusted (100 m resolution). The density was calculated by the authors of this study.
	(X13) Bus line density	The density of bus line in the buffer (km/km^2^)	WeChat official account: Lifangshujuxueshe (In Chinese) The original data was obtained from an open-source bus information query website.
Land use	(X14) Shannon’s Diversity Index (SHDI)	SHDI of the buffer	http://data.ess.tsinghua.edu.cn/ The original data is vector data of land-use status, and the SHDI is calculated by the author of this study.
	(X15) Normalized Difference Vegetation Index (NDVI)	NDVI of the buffer	http://www.resdc.cn/DOI),2022.DOI:10.12078/2022030801
	(X16) Floor–Area Ratio (FAR)	FAR of each buffer	WeChat official account: Lifangshujuxueshe (In Chinese) The original data are vector data of the building outline and the number of floors. The FAR is calculated by the authors of this study.
	(X17) Distance from the city center	Distance from community to city center (km)	Calculated by the authors of this study.
Society and Economy	(X18) Housing price	The average housing price of the buffer (Chinese Yuan)	WeChat official account: Dixuedashuju (In Chinese) (250 × 250 m resolution) https://mp.weixin.qq.com/s/kLO2wCDT9q1fcGPO4G8hPw

#### 3.1.2 Data processing

**Fig 1:**
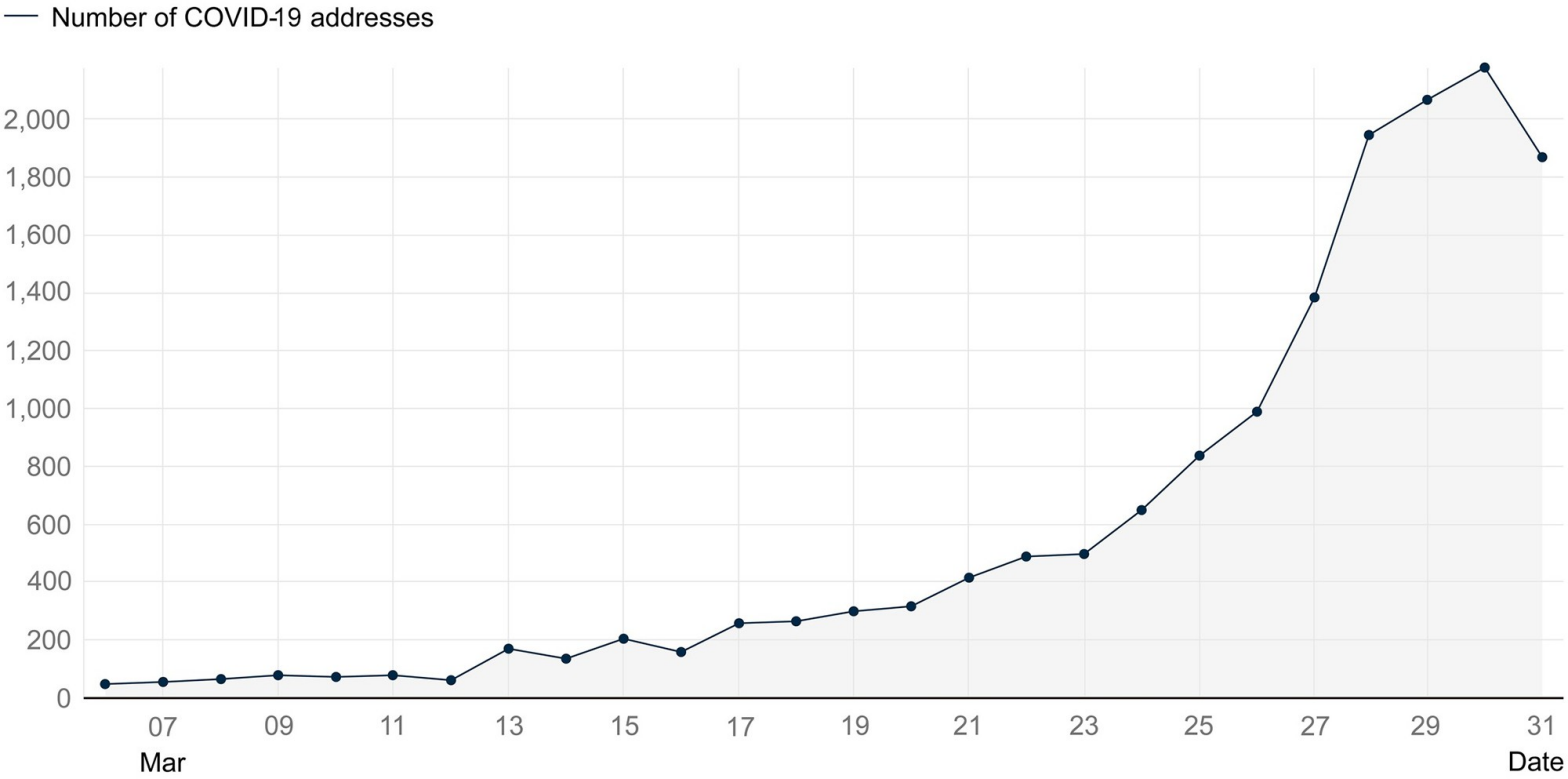
Number of COVID-19 addresses within the time range of the study. (Sources: Original data from Shanghai Municipal Health Commission, processed and visualized by authors according to the methods explained in the text).

*3*.*1*.*2*.*1 Processing of COVID-19 data and generation of COVID-19 addresses*. In the ensuing elaboration, statistics on reported COVID-19 addresses with COVID-19 cases (abbreviated as “COVID-19 addresses” for brevity) will be used the dependent variable to explore the associations between COVID-19 case distribution and the built environment, which is primary focus of this study. Since no matching reported information was available from March 1–5, cases with addresses reported from March 6–31 were considered for COVID-19 address extraction. Due to the a significant rise in cases, Shanghai Municipal Health Commission no longer provided the specific address of each infected individual separately from March 18, instead publishing collective addresses. Therefore, duplicate address data before March 18 were removed to mitigate potential errors in this study caused by the inconsistency in the caliber of official data statistics between the two periods. Ultimately, 15,593 data points representing the locations of the addresses of those who were infected with COVID-19, which can also be referred to as COVID-19 addresses, were identified throughout the entire area of Shanghai city. (Fig 1). The COVID-19 addresses in the study area were geocoded into points with geographic coordinates using the geocoding application programming interface (API) of the Gaode open platform(https://lbs.amap.com/). It is important to note that all data used in this study are sourced from open-access platforms, and the data collection and analysis methods adhere to the terms and conditions stipulated by the data sources. Specifically, the COVID-19 addresses used in this study do not disclose personal privacy or directly re-identify infected individuals.

*3*.*1*.*2*.*2 Processing of community data and generation of community buffer zones*. The study area in this paper encompasses communities within the main urban area of Shanghai, delimited according to boundaries specified in the Shanghai Urban Master Plan (2017–2035). Once the study area boundaries were defined, we processed the information collected about the community. Initially, we utilized the Gaode open platform’s API to geocode community addresses and obtain the spatial coordinates of each community’s geographic center. Due to the limitations in the precision of some raw data, some addresses were resolved to the same coordinates during geocoding process. Therefore, while filtering community coordinates within the main urban area boundaries, we ensured that duplicate coordinates were removed to prevent redundancy in later buffer zone creation. A total of 3,105 community geographic centers were identified and presented as points with coordinates extracted from their addresses. Considering the MAUP, the appropriate geographic scale was determined based on previous study by Liu et al. (2022) on COVID-19, community life circles, and the built environment in Wuhan, China [60]. Subsequently, these 3,105 screened communities were used to establish buffer zones with radii of 300, 500, and 800 m, corresponding to 5-, 10-, and 15-minute walking distances, respectively. The mean values of the variables selected in Section 3.2.1 within the buffer zones served as independent variables, while the count of COVID-19 addresses within buffer zones was taken as the dependent variable. In dense urban areas, where some communities have smaller areas, overlapping buffer zones may occur, potentially resulting in COVID-19 addresses being counted in multiple buffer zones simultaneously for analysis purposes.

It should be noted that the data collected from open platforms such as Gaode mostly use the GCJ-02 coordinate system, which is the mandatory encrypted coordinate system for domestic use in China. During data processing, all spatial data including COVID-19 addresses and communities, were uniformly converted from Gaode (GCJ-02) to the World Geodetic System (WGS-84) coordinate system before being used formally in the spatial dimension for more accurate results in subsequent analysis.

### 3.2 Model building

Boosted Regression Tree (BRT) is a technique that enhances the performance of a single model by fitting multiple models and combining them for predictions [61]. It integrates concepts and techniques from statistics and machine learning, focusing on algorithmic modeling and treating the data-generating mechanism as complex and unknown rather than starting from specific statistical models [62]. Unlike traditional statistical models, BRT does not require assumptions about the underlying statistical distribution of the response variable. This versatility allows BRT to accommodate various data types, including categorical, count, and continuous variables [63]. BRT models are widely used in the fields of ecology [64], geography [65], urban studies [66], disaster management [67], and epidemiology [68–70] to explore environmental impacts. Their advantage lies in their ability to identify non-linear relationships and assess the contributions of measurement indicators. Given the complex relationship between the built environment and COVID-19, a BRT model is well-suited for this study.

**Fig 2:**
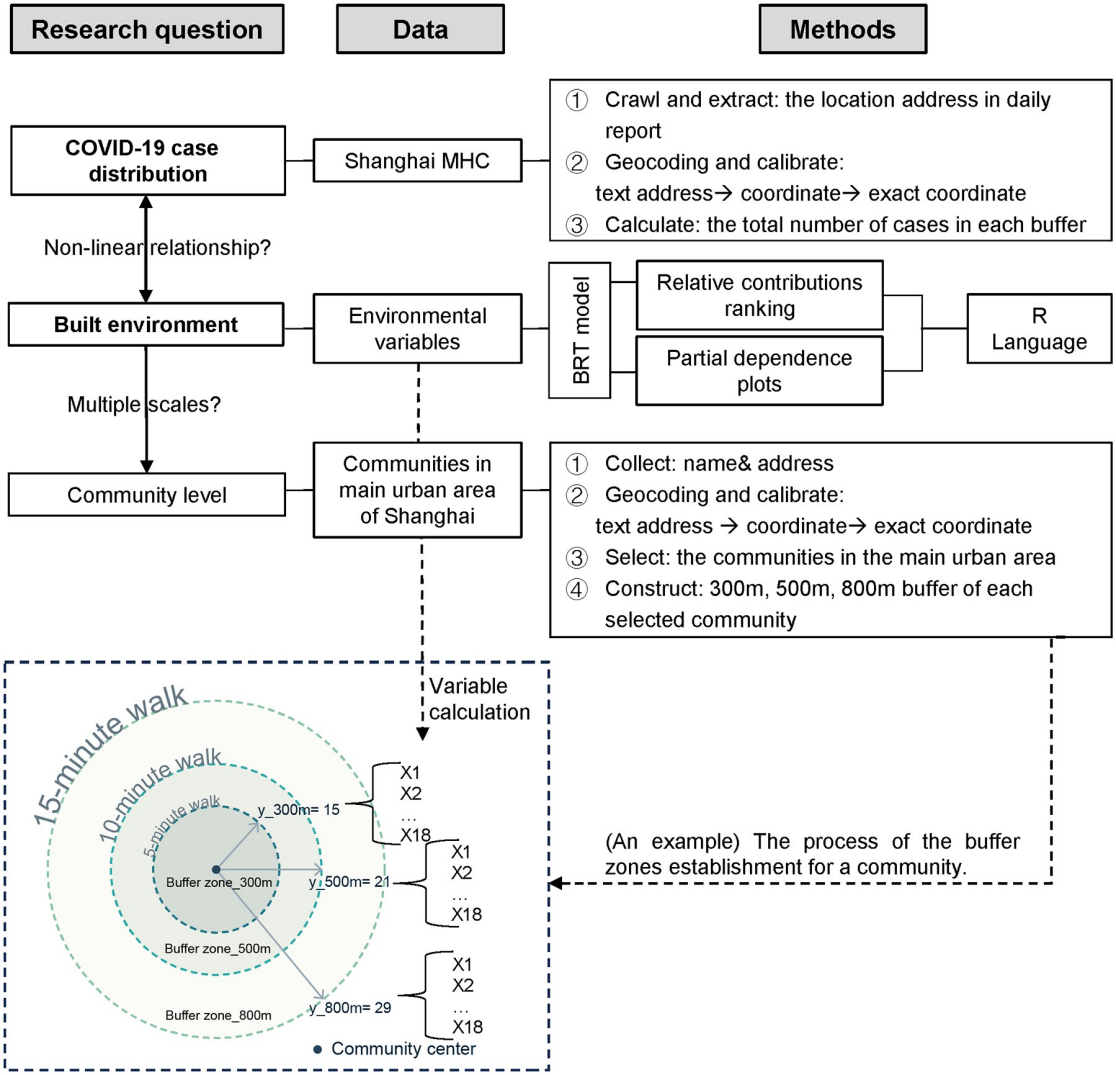
Workflow of the analysis and scale of buffer zones at the community level.

In this study, three BRT models were developed across three different scales using the gbm package (version 2.1.9) in the R programming environment. These models aimed to explore the non-linear relationship between the built environment and the number of COVID-19 addresses. The parameters tree complexity, learning rate, maximum trees, and bag fraction were set to 5, 0.005, 20,000, and 0.5, respectively. To evaluate the models, 10-fold cross-validation (CV) was employed for its computational efficiency and robustness in model evaluation [71]. The relative contribution of each variable to the total number of COVID-19 addresses at different scales was analyzed by using the split frequency importance method in BRT models. This method records how frequently each variable is used to split nodes across all trees in the model and the associated reduction in the loss function [63, 72]. These importance values were normalized to represent each variable’s percentage contribution to the model’s predictions. Differences in variable importance across scales were compared to understand their varying impacts. Based on the ranked importance scores provided by the BRT model, significant variables contributing to predicting the target variable were identified. This process facilitated the selection of important variables for further analysis. Subsequently, the BRT model was used to generate partial dependence plots for the top five influential variables identified in the previous steps. Finally, several metrics were used to assess each model’s performance and validity, including the results of 10-fold cross-validation. This comprehensive workflow is illustrated in Fig 2. The code used in this study can be found in S1 File.

## 4. Results

**Fig 3:**
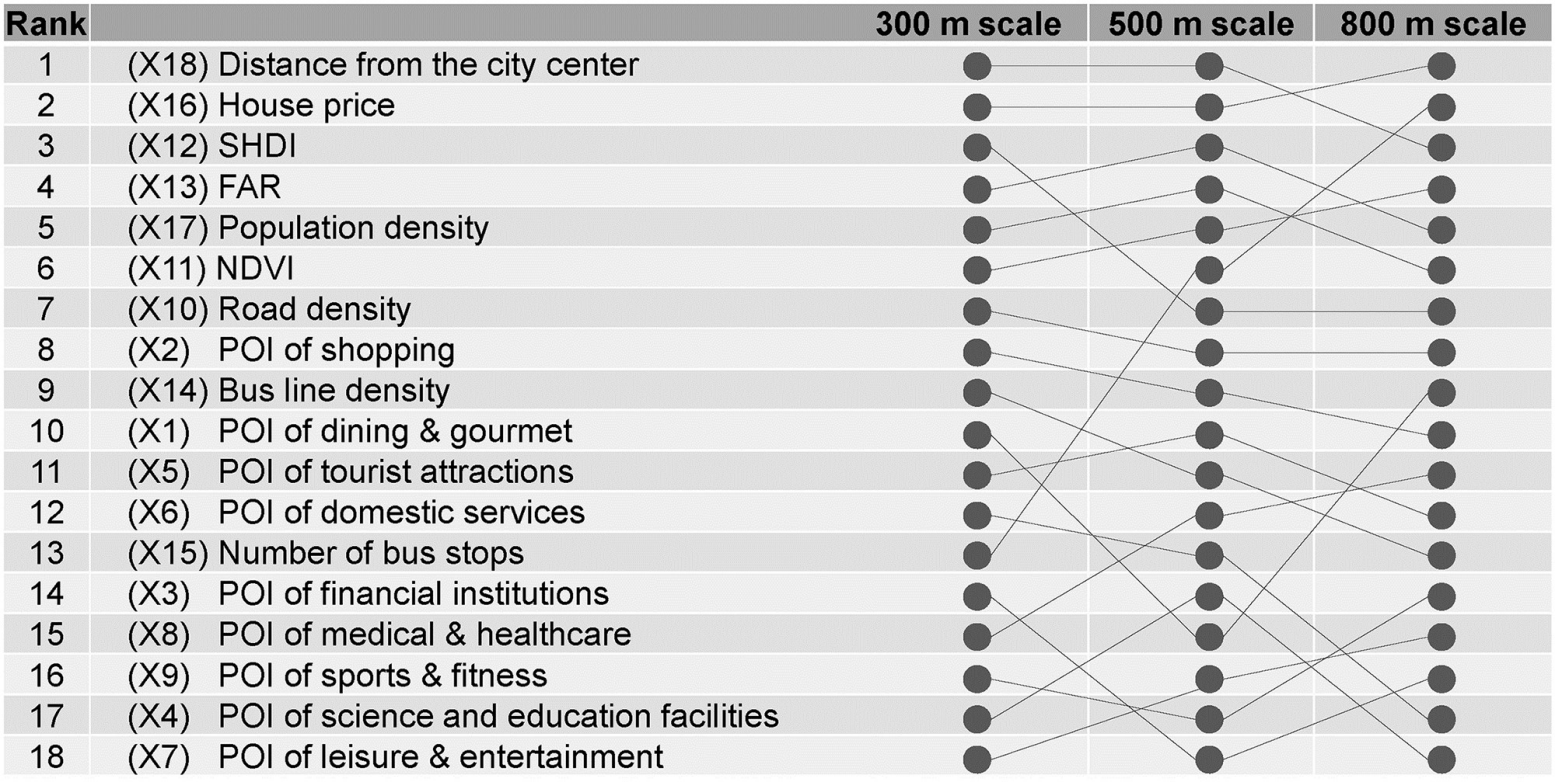
The ranking and the changes of the relative contributions (%) of each variable under different scales.

Table 2 and Fig 3 display the relative importance of each independent variable to the dependent variable in the models, along with their respective rankings. A higher ranking indicates greater importance of the variable in predicting the model’s outcomes. This ranking also illustrates how the contribution of each variable to the number of COVID-19 addresses varies across different scales. Consistent patterns were observed across the three scales. While the order of relative contribution rankings varied between scales, variables such as distance from the city center (X17), housing price (X18), and FAR (X16) consistently ranked among the top five in all scales. Housing price showed increasing contribution as the scale expanded, whereas SHDI showed decreasing contribution. Notably, distance from the city center ranked highest at the 500 m scale. Despite maintaining the same value across all three scales for each community (as it represents the distance from the city center), its performance in the model remains crucial for analysis. For instance, its contribution to the cumulative number of COVID-19 addresses notably decreased at the 800 m scale. In contrast, the contribution of the number of bus stops significantly increased at the 800 m scale.

**Table 2:** Variable’s relative contributions (%) ranking of each scale calculated by the BRT model.

300 m scale		500 m scale		800 m scale	
Variables	Contributions (%)	Variables	Contributions (%)	Variables	Contributions (%)
(X1) POI of dining & gourmet	3.45	(X1)	2.08%	(X1)	3.47%
(X2) POI of shopping	4.70	(X2)	4.32%	(X2)	3.12%
(X3) POI of financial institutions	2.30	(X3)	1.39%	(X3)	0.88%
(X4) POI of science and education facilities	1.68	(X4)	2.21%	(X4)	0.83%
(X5) POI of tourist attractions	3.43	(X5)	4.32%	(X5)	2.13%
(X6) POI of domestic services	3.24	(X6)	2.38%	(X6)	0.84%
(X7) POI of leisure & entertainment	1.33	(X7)	2.03%	(X7)	1.05%
(X8) POI of medical & healthcare	2.09	(X8)	2.40%	(X8)	2.85%
(X9) POI of sports & fitness	1.79	(X9)	1.57%	(X9)	1.17%
(X10) Road density	5.26	(X10)	5.77%	(X10)	4.55%
(X11) NDVI	6.99	(X11)	7.71%	(X11)	10.18%
(X12) SHDI	11.75	(X12)	6.80%	(X12)	6.92%
(X13) FAR	8.87	(X13)	9.18%	(X13)	8.31%
(X14) Bus line density	4.03	(X14)	3.87%	(X14)	1.96%
(X15) Number of bus stops	2.86	(X15)	6.83%	(X15)	13.09%
(X16) House price	12.05	(X16)	10.23%	(X16)	19.91%
(X17) Population density	7.80	(X17)	8.84%	(X17)	7.12%
(X18) Distance from the city center	16.38	(X18)	18.07	(X18)	11.63%

**Fig 4:**
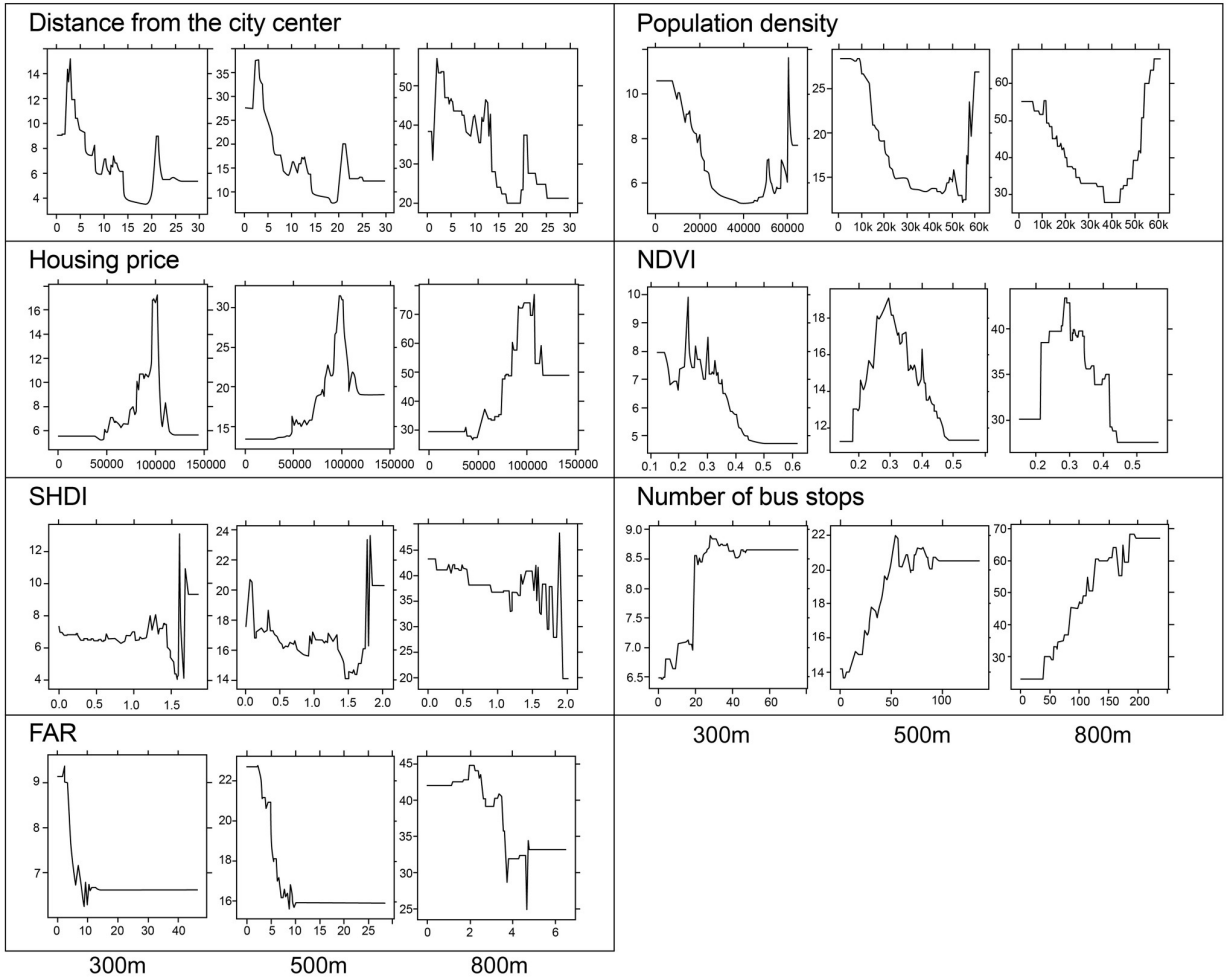
Partial dependence plots for the Top 5 influential variables in the BRT model. (X-axis: the value of the variable; Y-axis: the number of COVID-19 addresses).

Fig 4 depicts the partial dependence of the top five variables across different scales. The partial dependence plots reveal varying trends for each variable at different scales. The variable distance from the city center exhibits a consistent pattern across scales. Initially, the number of COVID-19 addresses remains high within a 5 km radius of the city center, decreases gradually, and then shows a rebound between 20–25 km. The housing price variable shows a similar trend at the 300 m and 500 m scales, with a notable increase in COVID-19 addresses in areas close to 100,000 yuan/m^2^, which becomes more pronounced at the 800 m scale. For the SHDI, at the 300 m scale, there are minimal fluctuations in COVID-19 addresses when SHDI is below 1.5, but a significant increase is observed when SHDI exceeds 1.5. At the 500 m scale, the number of COVID-19 addresses fluctuated, but the overall trend shows that COVID-19 cases decrease as SHDI increases below 1.5, with significant increases observed above 1.5. At the 800 m scale, smaller fluctuations and a downward trend are seen for SHDI values below 1.5, with larger fluctuations observed above 1.5. The FAR variable shows higher COVID-19 cases in its lower range, with most fluctuations occurring when FAR is below10. Regarding NDVI, values in the range of 0.2–0.3 tend to be associated with more COVID-19 cases. At the 800 m scale, there is a sharp increase in COVID-19 addresses with an increase of approximately 0.4.

Table 3 presents key metrics that evaluate the performance and validity of the BRT models. In terms of cross-validation, the CV correlation assesses how well the model predicts unseen data, with values closer to 1 indicating the better performance. The CV correlations for the three BRT models are 0.665, 0.842, and 0.855, indicating strong predictive capabilities across all models. RMSE and MSE values indicate the magnitude of deviation between model predictions and actual observations. Lower RMSE and MSE values suggest smaller prediction errors. Across the three models, deviations in model predictions are relatively small at the 300 m and 500 m scales. R^2^ measures how well the model fits the observed data, with values closer to 1 indicating a better fit. All three models demonstrate a high degree of fit.

**Table 3:** Performance metrics of three BRT models.

Model name	R^2^	CV Correlation	MSE	RMSE
BRT_300m	0.839	0.665	13.508	3.675
BRT_500m	0.939	0.842	21.596	4.647
BRT_800m	0.908	0.855	142.145	11.922

Note: MSE: Mean square error. RMSE: Root mean square error.

## 5. Discussion

There were notable findings in the study results and underlying mechanisms warrant discussion. Firstly, the BRT model revealed significant non-linear relationships among variables across different scales. The variable "distance from the city center" consistently ranked high across several scales. This may be attributed to factors such as higher density, and more complex human mobility patterns nearer to the city center, reflecting the intricate mechanisms of COVID-19 transmission. Furthermore, the non-linear results provided a clearer depiction of how COVID-19 addresses vary with different variables. While previous studies have generally acknowledged a positive correlation between population density and of COVID-19 transmission [73–75], this study elucidated non-linear relationships by examiningCOVID-19 addresses associated with low-, medium-, and high-density areas. These findings are pivotal for future research, particularly in densely populated mega-cities like Shanghai, where understanding such non-linear associations is crucial for effective pandemic management strategies.

Secondly, the research results were compared with conclusions drawn from previous studies. Previous research often concluded that a higher NDVI level indirectly mitigates the severity of the COVID-19 pandemic. For instance, Peng et al. explored the relationship between green space and COVID-19 incidence across 266 cities in China, finding a negative correlation between NDVI and COVID-19 incidence [76]. Similarly, Venter et al. suggested that green space contributes to increased social distancing, thereby indirectly reducing COVID-19 transmission [77]. However, this study not only found that higher NDVI values were associated with fewer COVID-19 cases but also pinpointed specific NDVI ranges linked to higher case numbers.

Regarding the SHDI, which reflects mixed land use, previous findings suggested that higher levels of mixed land use were associated with fewer COVID-19 cases, particularly at larger spatial scales (e.g., 15-min walking distance). In this study, at the 300 m scale, SHDI values below 1.5 showed minimal fluctuation in the number of COVID-19 addresses, with a downward trend observed as SHDI increased within the 500 m scale. However, when SHDI values reached 1.5 in both scales, COVID-19 addresses exhibited increased fluctuations. On a broader scale, Such as a 15-min walking distance, a higher degree of land-use mixture generally correlated with reduced the number of COVID-19 addresses in the area.

Regarding the FAR, it is important to note that the value range discussed, such as the node at FAR 10 in the results section, may exceed a typical neighborhood FAR values. This variation can be attributed to the inclusion of public buildings with higher FAR in certain buffer zones within the main urban area of mega-city Shanghai. Therefore, when interpreting the model, partial dependence plots within the range of typical neighborhood FAR values (generally below 10) more accurately reflect associations at the community level. Thus, the FAR partial dependence at the 800 m scale remains valuable for further interpretation, despite the BRT model at this scale not necessarily outperforming the other two models. For instance, the partial dependence indicates the corresponding number of COVID-19 addresses when FAR is below 6, a value not significantly higher than the general community FAR range. In essence, interpreting variables in the model should account for theses objective circumstances. Furthermore, in future research could weigh variables based on their contribution and evaluate different regions’ risk levels based on known non-linear relations. This approach could enhance preparedness for future public health emergencies akin to COVID-19.

This study had several limitations, notably uncontrolled confounding. The built environment encompasses various aspects of daily life beyond visible features like buildings, utilities, and transportation systems, and land use, unobserved socio-economic attributes of population groups including age, health status, income, add to the difficulty of understanding COVID-19 in terms of the equity [78]. These uncontrolled confounders across communities might also have associations with the number of COVID-19 infections. Future research will need to address uncontrolled confounding by considering equity and disparities in the built environment, differences in lockdown enforcement, COVID-19 susceptibility, case reporting rates, and initial/imported COVID-19 case numbers to further enhance the accuracy of the dependent variable. The interactions among the variables should also be considered. The mechanism of heterogeneity in different community types can also be further explored in future research.

In addition to committing to a more comprehensive selection of variables, improving the COVID-19 case data is crucial. Firstly, the research period for obtaining valid data was limited; in this study, it spanned only 25 days. Although the relevant model metrics have provided the evidence for good performance and validity, future studies should aim to collect data over longer periods to enhance our research. Second, the COVID-19 case distribution was characterized by COVID-19 addresses. Going forward, if the government can standardize its data release protocols and provide more accurate and open data while ensuring resident privacy protection, it would greatly benefit the advancement of relevant academic research.

## 6. Conclusions

The study utilized BRT models to explore the relationship between built-environment variables and COVID-19 across different scales. The main findings are as follows: (1) Relationships between built environment variables and COVID-19 case distribution vary across scales of analysis. The relative contribution of built-environment characteristics and their partial dependence on COVID-19 case distribution differ at various scales, highlighting varying strengths and directions of association. (2) Non-linear relationships between built-environment characteristics and the COVID-19 case distribution were identified. Key variables such as distance from the city center, population density, housing price, NDVI, SHDI, number of bus stops, and FAR demonstrated specific patterns in their partial dependence. Despite variability in model validity across scales, these non-linear results provide refined insights for pandemic response from the urban planning perspective at different scales.

Based on our results, several policy recommendations can be proposed for community-based healthy city construction in the post-pandemic era: First, consider important factors identified in urban planning responses to public health emergencies based on the relative importance ranking of variables. Second, incorporate critical nodes and thresholds identified through the marginal effect curves of non-linear relationships into urban planning considerations. Besides, different planning strategies should be adopted for variables associated with varying numbers of COVID-19 cases. For instance, in this study, an SHDI threshold of 1.5 serves as a critical demarcation point: neighborhoods below this threshold require distinct planning measures compared to those above it. Third, promote moderately compact neighborhood environments with ample land use, particularly focusing on enhancing green spaces in areas lacking them. Furthermore, enhance community-level healthcare facilities, especially in neighborhoods closer to the city center. These recommendations aim to optimize urban environments to better respond to future public health challenges, leveraging insights derived from the study’s findings.

## Supporting information

S1 File Code for the BRT model. (TXT)
